# Smoking Behaviors, Mental Health, and Risk Perceptions during the Beginning of the COVID-19 Pandemic among Mexican Adult Smokers

**DOI:** 10.3390/ijerph182010905

**Published:** 2021-10-17

**Authors:** Emily E. Loud, Katia Gallegos-Carrillo, Inti Barrientos-Gutiérrez, Edna Arillo-Santillán, Victoria C. Lambert, Luis Zavala-Arciniega, James F. Thrasher

**Affiliations:** 1 Department of Health Promotion, Education, and Behavior, Arnold School of Public Health, University of South Carolina, Columbia, SC 29201, USA; vlambert@email.sc.edu (V.C.L.); thrasher@mailbox.sc.edu (J.F.T.); 2 Epidemiological and Health Services Research Unit, Mexican Social Security Institute, Cuernavaca 62000, Mexico; kgallegosc13@gmail.com; 3 National Institute of Public Health, Mexico City 62100, Mexico; inti.barrientos@insp.mx (I.B.-G.); edna@insp.mx (E.A.-S.); 4 Department of Epidemiology, University of Michigan, Ann Arbor, MI 48109, USA; lzavalaa@umich.edu

**Keywords:** COVID-19, tobacco, smoking, risk perceptions

## Abstract

Mexico is one of the countries most affected by COVID-19. Studies have found that smoking behaviors have been impacted by the pandemic as well; however, results have varied across studies, and it remains unclear what is causing the changes. This study of an open cohort of smokers recruited from a consumer panel (*n* = 2753) examined changes in cigarettes per day (CPD), daily vs. non-daily smoking, recent quit attempts, perceived stress, depression, and perceived severity of COVID-19 at two points during the pandemic: March and July 2020. Differences in CPD between waves were estimated with Poisson regression using generalized estimating equations (GEE). Differences in perceived stress were estimated with linear regression using GEE, and differences in recent quit attempts, depression, and perceived severity of COVID-19 were estimated using separate logistic regression GEE models. Rates of depression were higher in July compared to March (AOR = 1.55, 95% C.I. 1.31–1.85), and the likelihood of recent quit attempt was lower in July compared to March (AOR = 0.85, 95% C.I. 0.75–0.98). There was no statistically significant change in CPD, daily smoking, or perceived stress. Perceived COVID-19 severity for oneself increased significantly (AOR: 1.24, 95% C.I. 1.02–1.52); however, the perceived COVID-19 severity for smokers remained constant. Our study suggests that as the COVID-19 pandemic expanded in Mexico, smoking frequency remained stable, and quit attempts decreased, even as adult smokers increasingly perceived infection with COVID-19 for themselves as severe. These results can aid in the development of health communication strategies to educate smokers about their risk for COVID-19, potentially capitalizing on concerns that stem from this syndemic of communicable and smoking-related non-communicable disease.

## 1. Introduction

An outbreak of atypical pneumonia was first reported in December of 2019 in Wuhan, China [1]. Caused by a novel coronavirus named severe acute respiratory syndrome coronavirus 2 (SARS-CoV-2), the accompanying illness now known as COVID-19 has reached pandemic proportions [2]. By September 2021, 229 million cases and 4.7 million deaths had been reported worldwide [3]. Risk factors for severe infection and death from COVID-19 include older age [4], comorbidities such as cardiovascular disease, diabetes, chronic respiratory disease, hypertension, cancer [5], obesity [6,7], and smoking [8,9]. Smoking is the leading cause of preventable death worldwide [10], and increases the risk for cancer, respiratory and cardiovascular diseases, inflammation, immunosuppression, diabetes mellitus, and diseases of nearly all organs in the body [11]. Many of these conditions exacerbate COVID-19, and studies have shown that smokers are at an increased risk for severe COVID-19 infection [12] and mortality [13]. It is also important to note that the COVID-19 pandemic has had a negative impact on mental health [14,15], increasing stress and diagnoses of anxiety and depression [16,17].

Given the evidence that the pandemic has resulted in an increase in stress, anxiety, and depression, and that these mental health conditions are associated with higher smoking rates, it could be expected that smoking rates would increase during the pandemic. However, studies of smoking behaviors during the pandemic have shown increases, decreases, and constant rates of smoking across a variety of populations [18,19,20]. Changes in smoking patterns, or lack thereof, have been attributed to stay-at-home orders impacting smoking behaviors [21], increases in stress and depression [22], and early, tobacco industry-associated studies indicating that nicotine may have been protective of COVID-19 infection [23].

As smokers are at a higher risk of severe COVID-19 [12,13], and considering the relationship between smoking and mental health, there is a need to understand how smoking behaviors, symptoms of depression and stress, and risk perceptions among smokers have changed over the COVID-19 pandemic period, and how these outcomes relate with one another. Examining smoking frequency and quit attempts are important because they predict cessation [24,25,26]. Examining associations between mental health and smoking behaviors can help illuminate the extent to which mental health may account for any changes in smoking patterns.

In addition to mental health, risk perceptions related to COVID-19 may also help explain smoking behaviors. Multiple health behavior theories, such as the Health Belief Model [27] and the Theory of Planned Behavior [28], posit that perceptions of the severity of and personal susceptibility to a disease or health condition are important determinants of the behaviors that protect one from getting the disease [29]. Indeed, these risk perceptions predict preventive behaviors in the context of infectious disease outbreaks [30], including handwashing, mask-wearing, and social distancing in the context of COVID-19 [31]. In the case of COVID-19 and smoking, smoking cessation would be considered a protective health behavior; therefore, examining risk perceptions may provide a richer understanding of why smokers may quit or reduce their smoking frequency due to the pandemic.

Optimistic bias, the tendency for individuals to underestimate their own risk for a health condition compared to others their age, can dampen the behavioral effects of risk perceptions [32]. Indeed, many smokers have optimistic biases regarding smoking-related diseases and nicotine addiction [33,34,35]. Nevertheless, smokers who perceive health consequences from smoking are more likely to quit [36,37]. Among the U.S. general population, there is evidence of optimistic bias in relation to risk perceptions of COVID-19 [38], as has been found for influenza [39]. Overall, a better understanding of COVID-19-related risk perceptions may also help us better understand smoking behaviors during the pandemic.

While all of these outcomes can help us understand smoking behaviors during the pandemic, the specific impacts of COVID-19 have varied from country to country, depending on a multitude of factors, including socioeconomic variation, testing rates, and policy [40]. Therefore, analyses involving specific countries may be needed to provide the most accurate picture. Mexico is one of the countries that has been most affected by COVID-19. Mexico reported its first case of COVID-19 on 28 February 2020 [41]. As of September 2021, Mexico reported 3.5 million cases, 272,000 deaths from COVID-19 [3], and has a case-fatality ratio of 9.4%, one of the highest in the world [42]. Mexico also has reported very low rates of testing, likely resulting in under-reporting of cases [43]. Furthermore, Mexico has a high prevalence of obesity [44], hypertension, cardiovascular disease, and diabetes [45], all of which are risk factors for severe COVID-19 infection. Of all confirmed COVID-19 cases in Mexico as of April 2021, 7.3% are among smokers [43].

In 2018–2019, prior to the pandemic, 17.9% of the adult population in Mexico smoked (7.7% daily; 10.2% nondaily). During the initial pandemic stage (August to November 2020), 16.8% of the population smoked (7.4% daily; 9.4% nondaily) [46]. Mental health conditions among Mexicans have increased over this period of time. Prior to the pandemic, 9.5% of Mexican young adults and 13.3% of Mexican older adults suffered from depression [47]. A study from March to April 2020 found that 50% of Mexican adults reported developing symptoms of depression and anxiety [48]. No studies have systematically assessed changes in mental health symptoms, risk perceptions, or smoking behaviors among Mexican smokers over the pandemic period.

The purpose of this study was to examine the trends in perceived stress and depression, smoking frequency, recent quit attempts, and the perceived severity of COVID-19 over time among adult Mexican smokers and dual users (i.e., smokers who also use e-cigarettes). We hypothesized that smoking frequency, recent quit attempts, perceived stress and depression, recent quit attempts, and the perceived severity of COVID-19 would increase from March to July 2020. Additionally, we anticipated that smokers would exhibit optimistic bias about the severity of COVID-19, but that this would decrease over the course of the pandemic, as its impact and gravity has increased and has been widely covered in the media.

## 2. Materials and Methods

### 2.1. Participants

Mexican smokers were recruited to participate in an open-cohort study through Kantar, an online commercial panel for marketing research, with study design details described elsewhere [49,50,51]. Eligible participants were 18 years or older and had smoked at least 100 cigarettes in their life and at least once in the last 30 days. At each survey wave, approximately 1500 people participated, with quotas used to ensure a range of educational attainment (at least 500 with high school education or lower) and use of e-cigarettes in the prior month (at least 500 participants), with replacement strategies used to replenish the sample and maintain sample size. Dual users (i.e., smokers who also use e-cigarettes) were oversampled to address key research questions in the parent study. The present study included data from two survey waves: the first from March 16–26 of 2020 and the second from July 16–28 of 2020. There were two analytic samples: Sample A included the full sample of eligible participants (March *n*=1395, July *n* = 1358, total *n* = 2753). Sample B included a subsample of participants who responded to COVID-19 perception items (March *n* = 606, July *n* = 1193, total *n* = 1799). Follow up from March to July was 55.6% (*n* = 834), which was expected given the study design.

### 2.2. Data Collection

The survey was administered online and took approximately 20–25 minutes to complete. All questions were administered in Spanish using questions from the International Tobacco Control (ITC) Smoking and Vaping survey [51,52] and the International Agency for Research on Cancer (IARC) [53]. Study procedures were approved by the Institutional Review Board and Ethics Committee of the National Institute of Public Health of Mexico (Ethical Approval Code: CI 1572).

### 2.3. Measures

#### 2.3.1. Dependent Variables

Depression was measured with two previously validated questions from the Patient Health Questionnaire-2 [54]: 1) ‘During the last 30 days, how often have you felt difficulties were piling up so high that you could not overcome them?’ and 2) ‘During the last 30 days, have you often been bothered by little interest or pleasure in doing things?’ with response options ranging from 1 to 5 (never, rarely, sometimes, often, very often). Participant scores ranged from 1 to 6 were dichotomized where a score of more than 3 indicated depression and a score less than or equal to 3 indicated no depression, for which the validity of this measure has been validated in the Mexican population [55].

Perceived stress was measured through asking respondents two items: (1) ‘During the last 30 days, have you felt confident that you can handle your life problems?’, and (2) ‘During the last 30 days, did you feel that things are going as you want?’ [56]. For each question, response options ranged from 1 to 5 (never = 5, rarely = 4, sometimes = 3, often = 2, very often = 1) and a perceived stress score was derived by adding the scores from the two items together to create a continuous variable ranging from 1 to 10, where a higher score indicated higher perceived stress.

The smoking-related independent variables were measured as follows: smoking frequency (daily [reference] or non-daily) and quit attempt within the last 4 months (yes or no [reference]). Daily smokers reported the number of cigarettes they smoked per day (CPD). Weekly smokers reported the number they smoked per week, which was then divided by 7 to calculate the number of CPD.

Perception of COVID-19 severity for oneself was measured with the following item: ‘Compared with other people your age, if you become ill with coronavirus, how severe do you think it would be?’. Perception of COVID-19 severity for smokers was measured with the following item: ‘In your opinion, if a smoker becomes ill with coronavirus, how would his/her smoking affect the severity of the illness? Would you say his/her smoking would make the illness…?’. Response options for both items (i.e., ‘much more severe’ = 1, ‘a lot more severe’ = 2, ‘a little more severe’ = 3, ‘equally severe’ = 4, ‘a little less severe’ = 5, ‘a lot less severe’ = 6, and ‘much less severe’ = 7) were dichotomized (i.e., 1–3 = ‘more severe’; 4–7 = ’equally or less severe).

These two questions were also used to derive a continuous measure of optimistic bias by subtracting the original response to perceived COVID-19 severity for smokers from perceived COVID-19 severity for oneself, which is in line with previous measurements of optimistic bias [57,58]. This measure was dichotomized where observations with a positive difference were classified as having ‘optimistic bias’ and those with a negative difference or a difference equal to 0 were classified as not having ‘optimistic bias’. When comparing the continuous measure and dichotomous variable of optimistic bias as an outcome, the results and their interpretation were consistent.

#### 2.3.2. Covariates 

Covariates included sociodemographic characteristics: age (18–29 [reference], 30–39, 40–49, 50+), sex (male [reference] or female), educational attainment (middle school or less [reference], some college/high school/technical school, university or more), and family income (<=8000 pesos [reference], 8001–15,000 pesos, 15,001–20,000 pesos, >20,000 pesos; approximate exchange rate: $20 pesos = $1 US dollar). Additionally, the smoking-related characteristics assessed included: type of user (exclusive cigarette user [reference]; sporadic dual user, uses e-cigarettes 1–2 days per week, frequent dual user, uses e-cigarettes 3 days a week or more), smoking frequency (daily [reference] or non-daily), quit intention within 6 months (yes or no [reference]), and quit attempt within the last 4 months (yes or no [reference]).

### 2.4. Statistical Analysis 

We assessed the descriptive statistics for all categorical variables and used chi-square tests to compare differences in frequencies between March and July samples. The full sample (Sample A) was analyzed for models including perceived stress and depression, smoking frequency, recent quit attempts, and CPD. The sub-sample (Sample B) was analyzed for perceived COVID-19 severity. All models to test for changes over time involved generalized estimating equations (GEE) to adjust for within-individual correlations for those who participated in both surveys. Separate logistic regression models were estimated for each dichotomous dependent variable (smoking frequency, recent quit attempt, depression, perceived COVID-19 severity, and optimistic bias). A Poisson regression model was estimated for analysis of CPD. A linear regression model was estimated for analysis of perceived stress. All models included an indicator for the survey wave (March = reference) and assessed multicollinearity using variance inflation factors, finding no evidence of any issues.

Models of perceived stress and depression included sex, age, educational attainment, family income, smoking frequency, recent quit attempts, and wave as covariates. For the smoking-related outcomes assessed, covariates were sex, age, educational attainment, family income, wave, perceived stress, and depression. We included stress and depression in order to determine whether they were likely to explain any changes we found in these outcomes. Models for perceived COVID-19 severity and optimistic bias included the covariates of sex, age, educational attainment, dual use status and frequency, smoking frequency, family income, and wave. To account for differences in age between the March and July waves and to further examine the relationship between age and all COVID-19 risk perception outcomes over time, we also estimated fully adjusted models that included interactions between age (18–39 vs. 40 and older) and wave. Analyses were conducted using Stata v.16 (StataCorp, Lakeway Dr., College Station, TX, USA]).

## 3. Results

### 3.1. Sample

Participant characteristics for Sample A and Sample B are shown in Table 1. We found no significant differences in participant characteristics between March and July waves in Sample A, the full sample. In Sample B, the sub-sample of participants who responded to COVID-19 perception items, participants in the July wave were older (*p* < 0.001) and were more likely to be daily smokers (*p* < 0.001) and to have recently tried to quit (*p* = 0.003).

### 3.2. Depression and Perceived Stress

Symptoms of depression from March to July are shown in Table 2. In March of 2020, 19.9% of participants reported symptoms of depression, which increased to 27% in July of 2020 (*p* < 0.001). After adjusting for sociodemographic variables and smoking characteristics, participants in July were significantly more likely to report symptoms of depression compared to participants in March (AOR: 1.55, 95% C.I. 1.31–1.85).

The results for perceived stress are shown in Table 3. The mean perceived stress score increased from 5.23 (SD = 1.88) in March to 5.35 (SD = 1.81) in July (Coeff = 0.12, 0.00, 0.24); however, this increase was not statistically significant in adjusted models (Coeff = 0.10, (−0.01, 0.22). 

### 3.3. Smoking Frequency and Quit Attempts 

The differences in CPD, daily smoking, and recent quit attempts from March to July are shown in Table 4. For the full sample, there were no significant differences in CPD or daily smoking in July compared to March. However, participants in the July survey were much less likely to report a recent quit attempt (AOR _March vs. July_= 0.84, 95% C.I. 0.74–0.96).

### 3.4. Perceived Severity of COVID-19 

**Figure 1:**
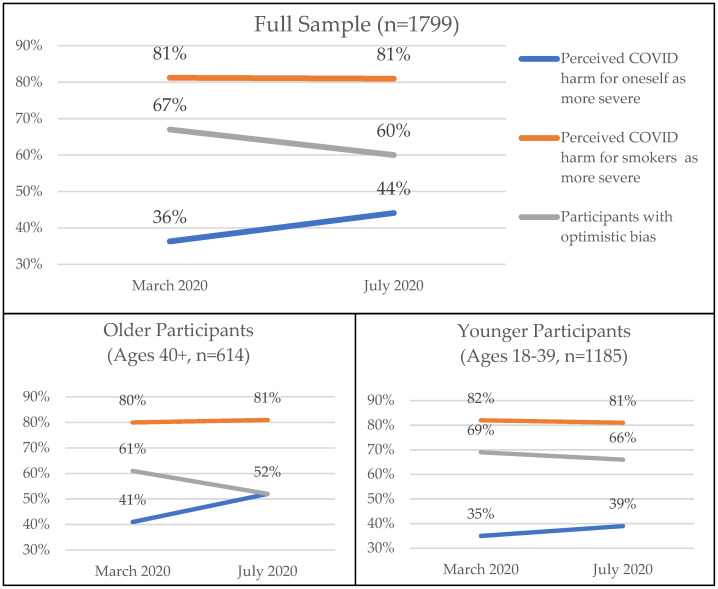
COVID-19 severity perceptions and optimistic bias by wave and between age groups among Mexican adult smokers and dual users (Sample B, 2020).

In March, 36% of participants perceived that COVID-19 would be more severe for them compared to other people their age, which increased to 44% in July (See Figure 1). In both waves, 81% of participants perceived that COVID-19 would be more severe for smokers than nonsmokers. The frequency of optimistic bias (i.e., perceiving severity for oneself as less than severity for smokers) decreased from 67% in March to 60% in July.

For older participants ages 40+ (*n* = 614), the increase in perceived COVID-19 harm for oneself increased from 41% to 52%. This increase for younger participants ages 18–39 (*n* = 1185) was from 35% to 39%. Similarly, optimistic bias in older participants decreased from 67% to 60% and in younger participants from 69% to 66%.

The prevalence of perceiving COVID-19 to be more severe for themselves than for others their age (see Table 5) was higher in July than in March 2020 (AOR _July vs. March_ = 1.24, 95% C.I. 1.02–1.52). Additionally, participants who held this perception were less likely to be male (AOR _male vs. female_ = 0.77, 95% C.I. 0.63–0.93) and more likely to be older (AOR _50 years old or older vs. 18–29_ = 2.07, 95% C.I. 1.52–2.81) and to be daily smokers (AOR _daily smokers vs. non-daily smokers_ = 1.45, 95% C.I. 1.18–1.78). 

There was no significant difference between March and July in the percentage of participants who perceived COVID-19 severity for smokers to be more severe than for nonsmokers. However, this perception was less likely among males than females (AOR = 0.74, 95% C.I. 0.57–0.95) and among those aged 30–39 compared to 18–29 (AOR = 0.68, 95% C.I. 0.50–0.92). 

Optimistic bias was not significantly different in March than in July. Optimistic bias was lower amongst all age groups older than the youngest participants (AOR age _30–39 vs. 18–29_ = 0.71, 95% C.I. 0.55–0.91; AOR _age 40–49 vs. 18–29_ = 0.67, 95% C.I. 0.49–0.91; AOR _age 50+ vs. 18–290_ = 0.43, 95% C.I. 0.31–0.58), frequent dual users compared to exclusive smokers (AOR = 0.64, 95% C.I. 0.46–0.89), and daily compared to non-daily smokers (AOR = 0.64, 95% C.I. 0.52–0.79). Participants with optimistic bias were more likely to be of higher educational attainment (AOR _some college/high school/tech school vs. middle school graduate or less_ = 1.41, 95% C.I. 1.00–2.00). We found that for all three outcomes, none of the interactions between age and wave were statistically significant.

## 4. Discussion

In this study of Mexican adult smokers over the early COVID-19 period (March to July 2020), depression increased, consistent with other evidence of the negative impact of COVID-19 on mental health [14,16,17]; however, perceived stress did not change over time. Furthermore, although CPD and daily smoking remained stable over time, attempting to quit decreased. In spite of the increase in depression, our results suggest that smoking behaviors remained relatively constant in the early COVID-19 period, as opposed to resulting in an increase in smoking.

We found no changes in smoking frequency during first several months of the COVID-19 pandemic, which is consistent with nationally representative data from Mexico showing no statistically significant changes in smoking frequency or daily smoking prevalence before and during the pandemic [46]. However, some studies from the USA have found variation in smoking patterns over the course of the pandemic depending on the population, with some reporting decreases in smoking during the pandemic [59,60,61] and others finding increases in smoking [62]. Nevertheless, tobacco industry reports in the USA indicate no change on overall volume of cigarette consumption during the pandemic [63]. Our inclusion of stress and depression variables as covariates did not appear to affect any estimates of smoking-related outcomes. 

Coupled with the stability of smoking frequency, our finding that smoking quit attempts decreased during the early stages of the pandemic should alert decision-makers to the importance of reinforcing and promoting access to tobacco cessation treatments. Smoking cessation for as little as 4 weeks can reduce the risk of COVID-19 infection and complications [64]. As such, decision-makers, researchers, intervention developers, and healthcare providers should consider emphasizing the COVID-related benefits of smoking cessation interventions, as the pandemic may serve as an important “cue to action”. After our data were collected, Mexico implemented a pictorial health warning label for cigarettes that illustrates and describes the increased severity of COVID-19 for smokers [65]. Future research should determine whether this strategy, which includes promotion of a cessation quitline, has had a meaningful impact on smoking behaviors, so that other countries—especially those with limited resources—might consider a similar, low-cost strategy.

Intervention development will likely need to consider how smokers perceived the risk for COVID-19. In our sample, the vast majority of participants believed that COVID-19 would be more severe for smokers, consistent with another study in the USA [66]. This perception remained stable from March to July, perhaps reflecting inconsistent media coverage of the relationship between COVID-19 and smoking. During this time period, coverage included contradictory content both around the severity of COVID-19 for smokers and the potentially protective effects of nicotine against contracting it, which came out of tobacco-industry sponsored research [23]. Nevertheless, we found a significant increase in perceiving COVID-19 to be more severe for oneself compared to others, which may reflect the growing number of COVID-19 cases over this early period of the pandemic. While this perception did not appear to translate into reductions in COVID-19-related optimistic bias, this bias was present in more than half of participants. This result aligns with other studies showing that risk perceptions of COVID-19 among the general population have increased over the course of the pandemic, with findings indicating potential optimistic bias general population samples in multiple countries [31,67].

In terms of specific characteristics associated with risk perceptions, while there was not a significant interaction between age and wave in adjusted models, older participants were more likely to perceive harm for themselves and less likely to exhibit optimistic bias compared to younger participants. Higher COVID-19 mortality among older populations has been highly increasingly publicized, which could explain this result [2]. Additionally, as the pandemic has continued to cause excess mortality, it is possible that perceptions are changing as participants witness the impact of COVID-19 on older populations. Female participants were more likely to perceive harm for smokers and for themselves, which is in line with previous research showing that female smokers tend to have higher smoking-related risk perceptions than male smokers [68,69]. In our sample, daily smokers were more likely to perceive greater COVID-19 harm for themselves and were less likely to have optimistic bias. This is consistent with other research showing that smoking frequency and risk perception for smoking-related diseases are positively correlated [36]. Indeed, more frequent smokers may have experienced more smoking-related health symptoms. We also found that smokers who use e-cigarettes more frequently (i.e., dual users) were less likely to exhibit optimistic bias. One potential explanation for this finding is that more frequent dual users are in the process of switching to exclusive e-cigarette use [70], perhaps because their perceptions of risk from smoking are relatively higher than exclusive smokers or less frequent dual users. This is an area for future research.

Although this study provides initial insights into how the initial stages of the pandemic impacted Mexican smokers’ mental health, smoking behaviors, and risk perceptions, it has some limitations. Our convenience sample was recruited from a non-probability sample that over-represents higher SES consumers, and we included quotas to oversample e-cigarette users. Hence, it is not clear how our results generalize to the broader population of Mexican smokers. Additionally, our sample of participants that responded to risk perception items was significantly older and were more likely to be daily smokers and report a recent quit attempt, which may have biased our results. Future studies should examine these variables in a representative sample of smokers. There were some limitations to our measures as well. Our assessment was limited to perceived severity of COVID-19, not perceived susceptibility, which can be an important influence on behavior, or other dimensions of risk perception (i.e., probability, worry and feeling unsafe) [71]. Future research should consider other dimensions of perceived risk, especially given news coverage of nicotine protecting against contracting COVID-19. Additionally, our measures of perceived stress and depression have good measurement properties [54,55,56] but involve relatively few items. More comprehensive measurement of these domains may be more sensitive to COVID-related states. Additionally, we did not assess whether participants had previously contracted COVID-19 prior to the survey; therefore, we were unable to account for this in our analysis, which may have impacted risk perceptions. Nevertheless, the incidence of COVID-19 was relatively low at the time of data collection and would be unlikely to seriously affect our findings. Future studies measuring COVID-19 risk perceptions should evaluate the influence of experience with COVID-19. Additionally, the incorporation of location and differential spread of COVID-19 over time is an area for future research.

## 5. Conclusions

Overall, this study provides evidence of how the early phase of COVID-19 pandemic impacted Mexican adult smokers. Our results show that depression increased, smoking frequency and intensity remained stable, and quit attempts declined. Furthermore, while the perceived severity of COVID-19 for oneself increased, optimistic bias around the potential severity of COVID-19 infection was stable but prevalent. Together, these results suggest that Mexican adult smokers may face more difficulties with smoking cessation during COVID-19, perhaps partly due to its impact on their mental health. As COVID-19 spreads and cycles in and out, interventions may need to be developed to increase smokers’ perceptions of the severity of COVID-19 for themselves, not just for smokers in general. Mexico recently introduced a cigarette pack warning label on COVID-19 severity among smokers [65], and its impact on smokers’ risk perceptions should be evaluated. It may also be important to develop smoking cessation interventions that recognize mental health issues related to COVID-19 and the apparent barriers they present for cessation. However, further research is needed to understand the influence of COVID-caused mental health issues on smoking cessation.

## Figures and Tables

**Table 1:** Sample characteristics among Mexican adult smokers, 2020.

	Sample A (Full Sample)			Sample B (Sub-Sample)		
Variables	March (*n* = 1395) %	July (*n* = 1358) %	*p*-Value *	March (*n* = 606) %	July (*n* = 1193) %	*p*-Value *
**Sex**						
Male	51	53	0.335	49	52	0.146
**Age group**						
18–29	31	30	0.494	44	31	<0.0001
30–39	31	31		31	30	
40–49	17	19		11	19	
>50	21	20		14	20	
**Educational attainment**						
Middle school graduate or less	11	9	0.199	11	10	0.454
Some college/high school/tech school	55	57		61	59	
College degree or higher	34	34		28	31	
**Household income ^a^**						
Less than 8000 MX monthly	23	25	0.563	30	25	0.221
8001 to 15,000 MX monthly	30	31		28	30	
15,001 to 20,000 MX monthly	16	17		14	17	
>20,000 MX monthly	27	24		23	23	
No response	5	5		5	5	
**Dual use status and frequency**						
Exclusive cigarette smoker	59	61	0.700	54	50	0.229
Sporadic dual user	26	25		34	37	
Frequent dual user	14	14		13	13	
**Smoking frequency**						
Non-daily	52	52	0.967	65	54	<0.001
Daily	48	48		35	46	
**Recent quit smoking attempt** **(Last 4 months)**						
Yes	43	40	0.070	53	60	0.003
**Intention to quit** **(Next month-6 months)**						
Yes	38	35	0.186	61	65	0.107

* *p*-values from Chi2 test for categorical variables among all respondents who participated in March and July. ^a^ MX = Mexican pesos; exchange rate for MX to USD is approximately $20 pesos = $1 US dollar.

**Table 2:** Symptoms of depression during the COVID-19 pandemic among Mexican smokers and dual users, 2020 (*n* = 2753).

Variables	Depression ^a^			
	No (<=3)	Yes (>3)	OR (95% C.I.)	AOR (95% C.I.)
**Wave COVID-19**	**%**	**%**		
March 2020, COVID-19, (*n* = 1395)	80.1	19.9 **	*Reference*	*Reference*
July 2020 COVID-19, (*n* = 1358)	73.1	27.0	1.48 (1.26, 1.74) **	1.55 (1.31, 1.85) **
**Gender**				
Female (*n* = 1321)	73.4	26.7	*Reference*	*Reference*
Male (*n* = 1432)	79.7	20.3	0.71 (0.59, 0.86) **	0.70 (0.58, 0.86) **
**Age**				
18–29 (*n* = 834)	69.1	30.9	*Reference*	*Reference*
30–39 (*n* = 850)	74.0	26.0	0.78 (0.62, 0.98) *	0.77 (0.60, 0.97) *
40–49 (*n* = 496)	83.9	16.1	0.44 (0.33, 0.60) **	0.43 (0.32, 0.59) **
50+ (*n* = 573)	85.3	14.7	0.39 (0.29, 0.52) **	0.37 (0.27, 0.51) **
**Education**				
Middle school or less (*n* = 282)	73.1	27.0	*Reference*	*Reference*
High school/technical studies/some college (*n* = 1544)	76.8	23.2	0.81 (0.60, 1.10)	0.88 (0.64, 1.21)
University or more (*n* = 927)	77.5	22.6	0.80 (0.58, 1.10)	0.93 (0.65, 1.33)
**Household income**				
≤8000 (*n* = 635)	70.4	29.6	*Reference*	*Reference*
8000–15,000 (*n* = 824)	76.5	23.5	0.74 (0.59, 0.94) *	0.80 (0.62, 1.03)
15,000–20,000 (*n* = 454)	78.9	21.2	0.66 (0.49, 0.88) *	0.73 (0.53, 1.00)
>20,000 (*n* = 704)	79.7	20.3	0.64 (0.49, 0.83) *	0.77 (0.56, 1.05)
missing (*n* = 136)	83.8	16.2	0.51 (0.31, 0.85) *	0.61 (0.36, 1.03)
**Smoking frequency**				
Non-daily (*n* = 1401)	77.2	22.8	*Reference*	*Reference*
Daily ≤ 5 CPD (*n* = 587)	78.2	21.8	0.95 (0.77, 1.22)	1.15 (0.91, 1.47)
Daily > 5 CPD (*n* = 695)	73.8	26.2	1.22 (0.98, 1.52)	1.66 (0.31, 2.11) **
**Recent quit smoking attempt (Last 4 months)**				
No (*n* = 1573)	78.9	21.1	*Reference*	*Reference*
Yes (*n* = 1110)	73.2	26.9	1.31 (1.10, 1.57) *	1.34 (1.10, 1.62) *

^a^ Logistic regression models using a generalized estimating equation (GEE) approach to consider repeated measures, adjusted included all variables presented in the table. * *p*-value < 0.05, ** *p*-value < 0.001.

**Table 3:** Scale of stress during the COVID-19 pandemic among Mexican smokers and dual users, 2020 (*n* = 2753).

Variables	Stress Variables (1–10) €		
Wave COVID-19	Mean (S.D.)	Coeff (Unadjusted)	Coeff (Adjusted)
March 2020, COVID 19, (*n* = 1395)	5.23 (1.88)	0.12 (0.00, 0.24) *	0.10 (−0.01, 0.22)
July 2020 COVID 19, (*n* = 1358)	5.35 (1.81)		
**Gender**			
Female (*n* = 1321)	5.52 (1.87)	*Reference*	*Reference*
Male (*n* = 1432)	5.08 (1.80)	**−0.44 (−0.59, −0.29)**	−0.36 (−0.51, −0.21) **
**Age**			
18–29 (*n* = 834)	5.72 (1.88)	*Reference*	*Reference*
30–39 (*n* = 850)	5.33 (1.84)	**−0.38 (−0.56, −0.19)**	−0.29 (−0.48, −0.10) *
40–49 (*n* = 496)	5.03 (1.78)	**−0.63 (−0.85, −0.41)**	−0.49 (−0.71, −0.27) **
50+ (*n* = 573)	4.84 (1.73)	**−0.85 (−1.06, 0.64)**	−0.72 (−0.94, −0.50) **
**Education**			
Middle school or less (*n* = 282)	5.63 (1.86)	*Reference*	*Reference*
High school/technical studies/some college (*n* = 1544)	5.31 (1.82)	**−0.31 (−0.56, −0.66)**	−0.20 (−0.44, 0.04)
University or more (*n* = 927)	5.14 (1.87)	**−0.52 (−0.78, −0.26)**	−0.19 (−0.46, 0.08)
**Household income**			
≤8000 (*n* = 635)	5.76 (1.79)	*Reference*	*Reference*
8000–15,000 (*n* = 824)	5.42 (1.77)	**−0.34 (−0.53, −0.15)**	−0.23 (−0.42, −0.03) *
15,000–20,000 (*n* = 454)	5.15 (1.86)	**−0.59 (−0.81, −0.37)**	−0.46 (−0.69, −0.23) **
>20,000 (*n* = 704)	4.86 (1.83)	**−0.87 (−1.07, −0.67)**	−0.64 (−0.87, −0.42) **
missing (*n* = 136)	5.05 (2.03)	**−0.68 (−1.03, −0.33)**	−0.56 (−0.91, −0.20) *
**Smoking frequency**			
Non-daily (*n* = 1401)	5.35 (1.85)	*Reference*	*Reference*
Daily ≤ 5 CPD (*n* = 587)	5.22 (1.82)	−0.11 (−0.30, 0.06)	−0.00 (−0.18, 0.17)
Daily > 5 CPD (*n* = 695)	5.21 (1.88)	−0.17 (−0.35, 0.00)	0.06 (−0.11, 0.24)
**Recent quit smoking attempt (Last 4 months)**			
No (*n* = 1573)	5.24 (1.88)	*Reference*	*Reference*
Yes (*n* = 1110)	5.35 (1.81)	0.05 (−0.08, 0.20)	0.00 (−0.13, 0.14)

Adjusted coefficients include all variables presented in the table. * *p*-value < 0.05, ** *p*-value < 0.001.

**Table 4:** Differences in daily smoking, recent quit attempts, and cigarettes per day (CPD) during the COVID-19 pandemic among Mexican daily and non-daily smokers, March 2020 and July 2020 (Sample A, 2020).

		**CPD ^1^** **(*n* = 2753)**	
**Wave**	Mean (SD)	IRR (unadjusted)	IRR (adjusted) ^b^
March 2020 (*n* = 1395)	4.34 (5.40)	*Reference*	*Reference*
July 2020 (*n* = 1358)	4.41 (5.42)	1.01 (0.95, 1.08)	1.01 (0.95, 1.08)
		**Daily smokers ^2^** **(*n* = 1282)**	
	%	OR (95% C.I.)	AOR ^a^ (95% C.I.)
March 2020 (*n* = 1395)	47.7	*Reference*	*Reference*
July 2020 (*n* = 1358)	47.8	1.08 (0.98, 1.20)	0.99 (0.88, 1.11)
		**Recent quit attempt ^3^** **(*n* = 1110)**	
	%	OR (95% C.I.)	AOR ^a^ (95% C.I.)
March 2020 (*n* = 1395)	43.1	*Reference*	*Reference*
July 2020 (*n* = 1358)	39.6	0.83 (0.73, 0.94) *	0.85 (0.75, 0.98) *

^1^ CPD is the average number of CPD among all respondents (daily and non-daily smokers). Generalized estimating equations (GEE) with Poisson distribution and log link function (IRR) were used to adjust for within-individual correlations in study variables for participants with repeated assessments. Adjusted IRR, by age, sex, educational attainment, family income and wave. * *p*-value < 0.05, ** *p*-value < 0.001; Dependent variables: ^2^ smoking frequency (daily vs. non-daily *n* = 1401 [reference]), ^3^ recent quit attempt vs. no recent quit attempt (*n* = 1573 [reference]). Logistic regression models using a generalized estimating equation (GEE) approach to consider repeated measures, models adjusted for by sex, age, educational attainment, family income and wave. ^a^ Logistic regression models using a generalized estimating equation (GEE) approach to consider repeated measures, ^b^ Linear regression models using a generalized estimating equation (GEE) approach to consider repeated measures.

**Table 5:** Independent correlates of COVID-19 severity variables among Mexican adult smokers (Sample B, 2020).

Variables	COVID-19 Severity for Oneself AOR [95% CI] ^a^	COVID-19 Severity for Smokers AOR [95% CI] ^a^	Optimistic Bias AOR [95% CI] ^a^
**Sex**			
Female	Ref	Ref	Ref
Male	0.77 (0.63–0.93) *	0.74 (0.57–0.95) *	1.06 (0.87–1.30)
**Age group**			
18–29	Ref	Ref	Ref
30–39	1.03 (0.80–1.33)	0.68 (0.50–0.92) *	0.71 (0.55–0.91) *
40–49	1.25 (0.92–1.68)	0.86 (0.58–1.27)	0.67 (0.49–0.91) *
≥50	2.07 (1.52–2.81) **	0.80 (0.54–1.19)	0.43 (0.31–0.58) **
**Educational attainment**			
Middle school graduate or less	Ref	Ref	Ref
Some college/high school/tech school	0.90 (0.64–1.27)	1.17 (0.79–1.75)	1.41 (1.00–2.00) *
College degree or higher	0.89 (0.60–1.31)	1.26 (0.80–2.00)	1.44 (0.97–2.14)
**Dual use status and frequency**			
Exclusive cigarette smoker	Ref	Ref	Ref
Sporadic dual user	1.23 (0.99–1.54)	1.03 (0.79–1.36)	0.84 (0.67–1.04)
Frequent dual user	1.12 (0.81–1.55)	0.79 (0.54–1.15)	0.64 (0.46–0.89) *
**Smoking frequency**			
Non-daily	Ref	Ref	Ref
Daily	1.45 (1.18–1.78) **	1.04 (.81–1.34)	0.64 (0.52–0.79) **
**Wave**			
March	Ref	Ref	Ref
July	1.24 (1.02–1.52) *	1.01 (0.78–1.30)	0.87 (0.71–1.07)

Dependent variables: perceived COVID-19 severity for oneself (more severe vs. equally or less severe [reference]), perceived COVID-19 severity for smokers (more severe vs. equally or less severe [reference]), and optimistic bias (yes vs. no [reference]). Logistic regression models using a generalized estimating equation (GEE) approach to consider repeated measures; ^a^ Models adjusted for all variables in the table. Adjusted by sex, age, educational attainment, dual use status and frequency, smoking frequency, family income, and wave. * *p*-value < 0.05, ** *p*-value < 0.001.

## Data Availability

Requests for data can be made to the primary investigator.
